# How to Paint Cemented Walls

**Published:** 1871-07

**Authors:** 


### How to Paint Cemented Walls.

Most painters, says Dr. H. Fruhling, who paint cemented walls, wash, previously, the surface with diluted acid, to remove the light patches which generally can be observed on newly erected and cemented walls, and affirm that only in such cases, that is, after restoring an even and uniform tint, can the work be finished successfully. This observation is not without foundation. The light patches, which the artisan calls *saltpetre*, consists often of carbonate of lime superficially adhering to the wall, and of real efflorescences of nitrates, which are formed, especially when the cement is laid in spring on the old and still frozen wall. In warm weather the walls condense the moisture, assisted by the hygroscopic property of fresh cement, and dissolve the caustic lime, which never is wanting in it, together with the salts that arrive at the outside from the interior. The carbonate of lime, into which, after the drying of the wall, the caustic lime is converted, remains mixed with the salts of the wall as a very light coating, hardly adhering to the surface, and presenting a great obstacle to the incorporation of the paint with the wall.

But nothing favors more the efflorescences on an old structure, than a fresh coating with cement. It can always be predicted with certainty, that a frost-like appearance on the outside indicates a wall containing saltpetre, the formation of which is only interrupted with the drying of the cement; and if the latter is inferior, those efflorescences will cause capillary cracks and pores, destroying, of course, the adhesion of the paint to the wall.

In washing such wall with diluted acid, those thin covers of carbonate of lime and salts are removed, and the surface, which a cement exhibits that has been prepared with too small an admixture of sand, is rendered of a suitable roughness, on which the paint sticks much better.

Carbonate of ammonia, however, has been found a far superior wash for the preparation of cemented walls than diluted acids, and the salt, which by long exposure to the atmosphere, has fallen into powder, may be used with advantage. If the cemented wall, having been left to itself for about twenty days, is brushed with a solution of this salt, (3 ounces in 10 quarts cold water,) the surface, after drying, will appear of an even grayish tint, and be exceedingly well adapted for holding the paint. The caustic lime, which was immediately under the out covering, has to be converted into carbonate of lime, and small water-like points, consisting of crystals of calcspar, unite the outside with the cement. The paint is readily and evenly taken up by this surface, which possesses in a remarkable degree, durability against frost and rain.

In case, however, washing with acid is preferred, dilute sulphuric acid is best, inasmuch as it forms with the lime an insoluble and not hygroscopic compound.—*Druggist's Circular.*

---

INFANT FEEDING.—An infant, for which the mother had no milk, and which they were attempting to bring up by hand, was shown to me when a few weeks old. It was puny, weak, and sickly. It always cried when an attempt was made to feed it, and could not hold up its head, which hung on one side from weakness. On inquiry, I found that it was fed on gruel, made of *fine* flour, mixed with unboiled milk, and heavily sweetened with brown sugar; and that latterly, to still its peevishness and cause it to sleep, a small quantity of *rum* was added to this. The sugar was given to prevent costiveness which, otherwise, it suffered from. It was acknowledged that the child was getting worse. "Put the sugar in your own tea," said I; "throw the rum out of the door, and send up your daughter to me immediately for a bowl of whole meal wheaten flour, the same as my own bread is made of." This I directed them to make into gruel, thus: "With a tablespoonful of this meal, and a pint of pure water, make a thin gruel, which should be boiled about fifteen minutes, and then about a pint of new milk fresh from the cow should be added;" the milk being of course unboiled, as before. These directions being followed, and the child being fed accordingly, in a week there was visible improvement, at the same time that red blotches, like those on the face of a drunkard, began to appear on the infant's face. All costiveness had now gone. At the end of six weeks from the commencement of change of diet the flesh of the child was firm and hard, its skin clear and bright, and it was perfectly good-tempered and quiet. Its weight, too, was about double what it was a few weeks before. The red blotches on the child's face, which appeared after the spirit was given up, were to be attributed to its constitution having gained strength by that time from its food sufficient to throw out the poisonous spirit, and they soon went away altogether. The infant is now at least fully as strong as the generality of children of the same age.—*Herald of Health.*

